# Supplementary material for: Polarization structured light 3D depth image sensor for scenes with reflective surfaces
Source: Nat Commun. 2023 Oct 27;14:6855. doi: 10.1038/s41467-023-42678-5 (PMC10611810; doi:10.1038/s41467-023-42678-5)
Supplement: Supplementary file 1 — Supplementary Information [file 41467_2023_42678_MOESM1_ESM.pdf]

Supplementary Information: Polarization Structured Light 3D Depth Image Sensor for Scenes with Reflective Surfaces

Xuanlun Huang[a,b,#], Chenyang Wu[a,b,#], Xiaolan Xu[b], Baishun Wang[b], Sui Zhang[b], Chihchiang Shen[b], Chiennan Yu[b], Jiaxing Wang[b], Nan Chi[a,c], Shaohua Yu[a,c], Connie J. Chang-Hasnain[a,b,*]

[a]*School of Information Science and Technology, Fudan University, Shanghai 200433, China*
[b]*Berxel Photonics Co. Ltd., Shenzhen 518071, China*
[c]*Peng Cheng Laboratory, Shenzhen 518055, China*

*Corresponding author
Email address: connie.chang@berxel.com (Connie J. Chang-Hasnain)
#These authors contributed equally to this work

I. Polarization characteristics of the HCG-VCSEL array.

As mentioned in the main text, HCG VCSELs have exhibited a very high polarization selection ratio independent of operating temperature or drive conditions, which can be even up to 30 dB [1, 2] with precise fabrication. Polarization selection ratio is defined by the ratio of power in TE and TM polarization. It can also be characterized by the orthogonal polarization suppression ratio (OPSR) curve, where

$$\text{OPSR} = 10\log(\frac{P_{\text{TE}}}{P_{\text{TM}}}) \tag{1}$$

Here, we compare the OPSR results of the HCG VCSEL array and the single HCG VCSEL in the same production patch in order to show that the VCSEL array possesses similar polarization characteristics as the single VCSEL. In Fig. S1a, we show the OPSR and power curve as the function of working current, where solid lines are the results of HCG-VCSEL array and dot lines are the results of DBR-VCSEL array. We can see that as the current increases, the OPSR of HCG-VCSEL array reaches the plateau at around 17 dB, meaning the output light of the HCG-VCSEL array possesses polarization. However, as for the DBR-VCSEL array, it keeps around 0 dB, which has no polarization. From the power curves of two types of arrays, we can see that the top HCG does not affect the output power much, making the HCG-VCSEL array suitable for the TX source. In Fig. S1b, we also show the OPSR curve and power curve of a single HCG VCSEL in the same fabrication patch. We can see that the OPSRs of the VCSEL array and the single VCSEL are close and the power obeys the multiple relation, which means the array almost has no impact on the original characteristics. In this patch, because of the fabrication error, the OPSR is not as high as that of [1, 2]. But this polarization ratio is sufficient to prove its superiority in the application of 3D imaging, as will be demonstrated in the main text. In addition, in order to control variables, we set the current of the PSL 3D sensor to be 1.5 A at each experiment, which means the output power is around 2.2 W. The driving condition is pulsed current with 33 milliseconds in period and 0.03 to 0.13 in duty cycle, namely 1 millisecond to 4.3 milliseconds in pulse width.

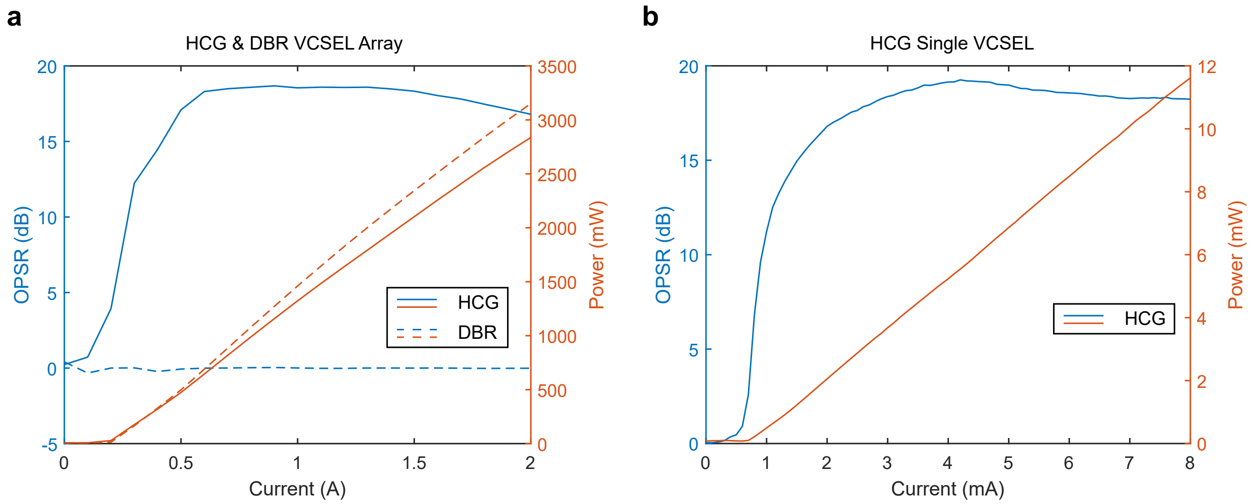

Figure S1: **OPSR and power curve of VCSELs.** **(a)** OPSR and power curve of HCG and DBR VCSEL arrays. **(b)** OPSR and power curve of a single HCG VCSEL.

II. Far-field of the dot-array structured light

As described in the Methods of the main text, the PSL 3D sensor produces dot-array structured light with over $30,000$ points, whose far-field distribution is shown in Fig. S2. This is achieved by the duplication of the VCSEL source array. As seen from Fig. S2, each block is a far-field projection of a VCSEL array, containing 364 randomly distributed points. Then this projection is duplicated to 11×9 diffraction orders uniformly by the diffraction optical element, enabling the generation of over $30,000$ dots in total. Note that the dot-array structured light possesses high polarization selection ratio. It means that these far-field points maintain the same polarized direction as the VCSEL source. If we put a polarizer whose polarized direction is orthogonal to the VCSEL on top of the TX, the far-field dots can be eliminated completely.

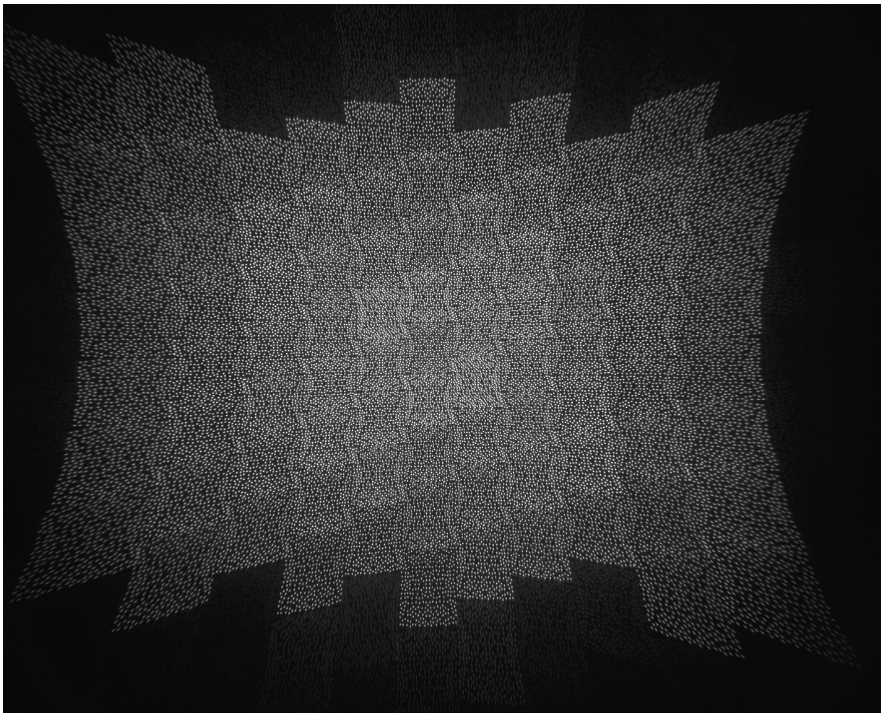

Figure S2: **Dot-array structured light.** This far-field distribution is captured with an industrial infrared camera at 40 cm.

III. Depth images comparison in a scene without glass

According to the Fresnel equation, the light reflected from the reflective surfaces maintains the same polarized direction as the incident structured light, while those from the diffuse surfaces have omnidirectional directions. This property can be best used to differentiate two types of reflecting surfaces. In Fig. 2, 4 and 6 of the main text, we have demonstrated that for a scene with the reflective surfaces, there is a sharp contrast between the depth channels from two polarization settings, which is an important cue for the determination of the reflective surfaces. Here, we provide the depth images in a scene without glass to support this method. In this scene (Fig. S3a), a cardboard box with its front surface removed is placed in front of the PSL 3D sensor. Inside the box, there is a box of gloves and a metal breadboard. We obtain two depth images (Fig. S3b and S3c) from two polarization settings, i.e., polarization 0° and polarization 90°. In Fig. S3b, we show the depth image in polarization 0°, which is obtained by setting both the TX and RX of the PSL 3D sensor in TM polarization. As for Fig. S3c, this is the depth image in polarization 90°, which is obtained by setting the TX in TM polarization and the RX in TE polarization. From the results, we can see that for a scene without glass, there is no sharp contrast between two polarization settings in the depth channel.

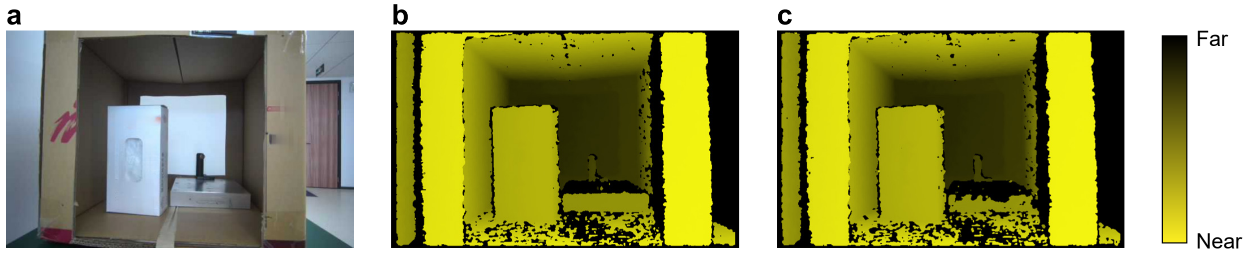

Figure S3: **Depth images comparison in a scene without glass.** **(a)** Scene of a cardboard box with its front surface removed. Inside the box, there is a box of gloves and a metal breadboard. **(b)** Depth image from the PSL 3D sensor, obtained by setting both the TX and RX in TM polarization. **(c)** Depth image from the PSL 3D sensor, obtained by setting the TX in TM and the RX in TE. The yellow-black color bar applies to both **(b)** and **(c)**.

IV. Fitting in the world coordinate

As shown in the step 2 of Fig. 2 in the main text, the extracted glass points are transformed to the world coordinate for fitting. Here we explain why it needs to fit in the world coordinate. We take the balcony glass #2 scene in the first column of Fig. S14 as an example. In Fig. S4a, we show the depth point cloud in x-z view. Because the depth point cloud originates from the depth image, it uses pixel as x unit and millimeter as z unit. In this subfigure, the glass points, which are extracted by the subtraction method as illustrated in step 1 of Fig. 2, are highlighted in red and the rest points are shown in grey. We can see that the glass frame (encircled by the white dashed line) is curved in the depth coordinate. This is because the internal parameters $\{f_x, f_y, c_x, c_x\}$ of the infrared camera will transform the point cloud coordinate to make it appear properly in the depth image. If we fit the glass plane based on the extracted glass points directly in the depth coordinate, it will deviate seriously from the glass frame, which is shown in Fig. S4b. Thus, the extracted glass points need to transform to the world coordinate. In Fig. S4c, we show the transformed point cloud in the world coordinate, where the glass points are also highlighted in red. In the world coordinate, both the x and z axis are in unit of meter. We can see that the glass frame is straight in this subfigure, which accords with the physical intuition. Then, the fitting can be employed under this coordinate and the result in Fig. S4d reveals that the red fitted plane matches well with the straight glass frame. At last, in Fig. S4e, the fitted and completed point cloud data is converted back to the depth coordinate, showing the correct result of our method.

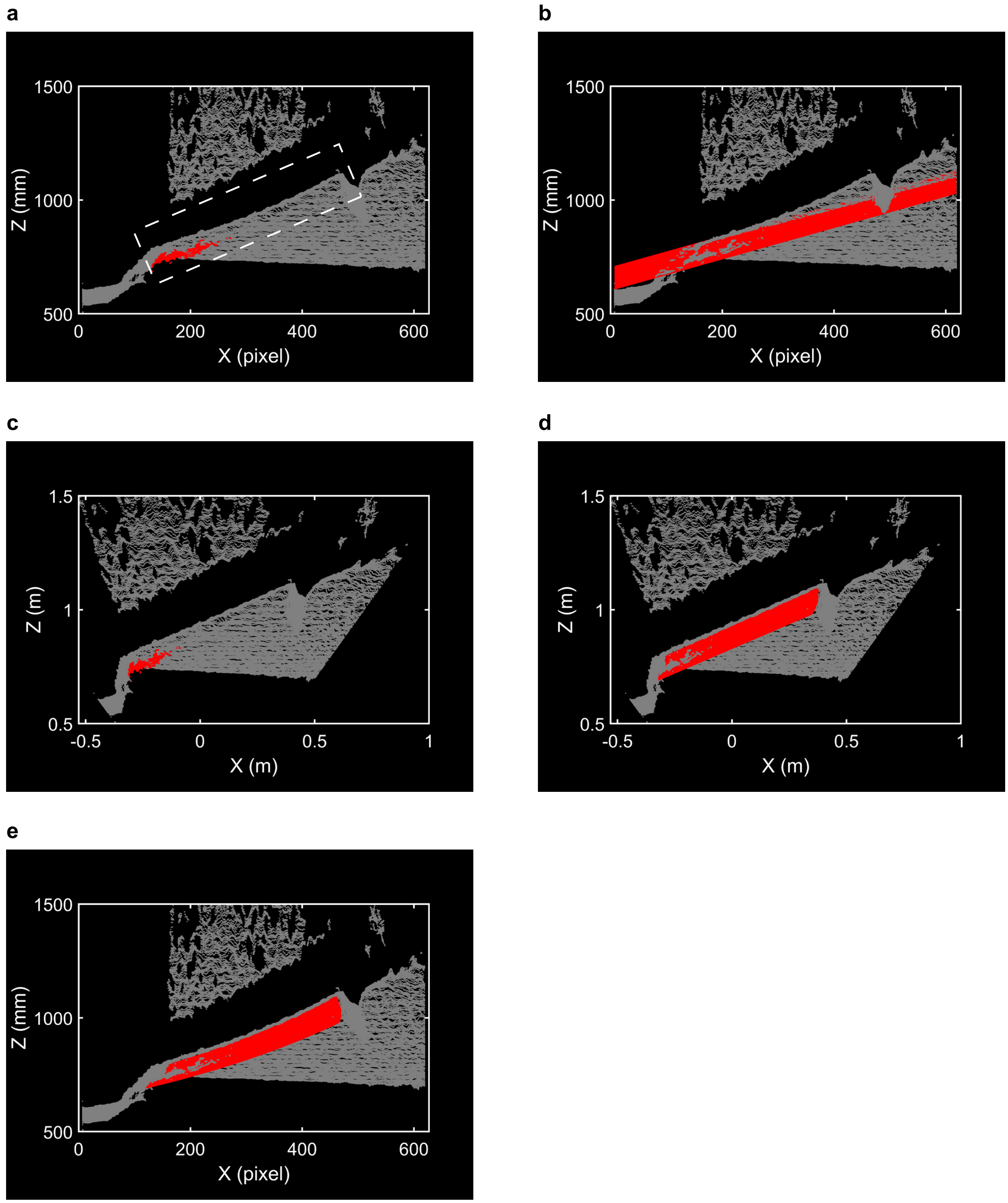

Figure S4: **Point cloud results in different coordinates.** **(a)** The original point cloud in the depth coordinate. The straight glass frame is curved in this coordinate. **(b)** Fitting directly in the depth coordinate. The fitted glass plane is shown in red. **(c)** Original point cloud in the world coordinate. **(d)** Fitting in the world coordinate, where the red fitted plane accords well to the straight glass frame. **(e)** The fitted point cloud that is transformed back to the depth

coordinate. Note that in the depth coordinate, x axis is in pixel unit and z axis is in millimeter unit while x and z axes are both in meter unit in the world coordinate.

V. Analysis on the influence factors of seeing through the reflective surface

In this section, we analyze the other three influence factors of seeing through the reflective surface. In Fig. S5, we show the results of PSL 3D sensor incident angle. In this experiment, we test three detecting angles, i.e., $-30°$, $0°$, $30°$ and their pictures of scene are shown in the first column of Fig. S5a, S5b, S5c. For each angle, the objects behind the glass are fixed to 0.5 m away and the distance from the PSL 3D sensor to the middle of glass is fixed to 0.8 m. Due to different incident angles, the position of the detected glass depth is also different. At $-30°$, the glass appears on the left of the depth map and does not overlap with the ball and cabinet, as shown in polarization $0°$ (Pol $0°$) of Fig. S5a. When the sensor is set to polarization $90°$ (Pol $90°$), the glass depth on the left can be eliminated, leaving only the ball and cabinet. When the detection angle is changed to $0°$, the glass depth appears around the ball at polarization $0°$, and similarly this glass information is eliminated at polarization $90°$. At $30°$, the detection and elimination of glass appears on the right side of the depth map. We can see that the different incident angle does not affect the function of seeing through the glass.

In Fig. S6, we show the results of different distances of objects behind glass. In this experiment, the distance from the sensor to the glass is set to 0.8 m and the detecting angle is $0°$. Then we change the distances of objects behind glass from 0.3 m to 0.7 m, and their results are shown in Fig. S6a, S6b, S6c, respectively. When the ball is close to the glass, more depth information about the ball is detected in the middle (white dot line in polarization $0°$ of Fig. S6a). As the distance increases to 0.5 m, the area of the ball decreases, and instead the glass in the middle is detected (white dot line in Fig. S6b). At 0.7 m, the middle is almost all the glass information (white dot line in Fig. S6c). At these three different distances, we can see that at polarization $90°$, the glass depth all can be eliminated. In the next experiment, we also discuss the effect of the density of objects behind glass.

Fig. S7 is the result of different densities of objects behind the glass. In this experiment, the distance of the PSL 3D sensor is 0.8 m, the distance of the rear object is 0.5 m, and the detecting angle is $0°$. When the density is low (Fig. S7a), the glass depth occupies most of the middle in polarization $0°$. When the density is in medium level (Fig. S7b), because the reflection of the rear object is stronger, part of the glass depth is replaced by the rear ball and cabinet. When the density becomes high (Fig. S7c), only the glass between the ball and the box can be detected. For all these three different levels, the part of glass can still be removed in polarization $90°$, leaving a clear depth

map of objects behind.

Although the density of objects behind affects the glass depth that we can detect, we can get enough depth information of glass by varying the distance and angle as demonstrated in the experiments of first three influence factors. Moreover, different experiments have shown that the glass depth can be eliminated at polarization 90°. This benefits from our specially designed HCG-VCSEL array, which allows the depth information belonging to the reflective surface to retain its original polarization characteristics.

Meanwhile, we also compare the sensor based on DBR VCSEL in Fig. S8. It can be seen that the glass depth area in polarization 0° and polarization 90° is similar, and the function of elimination cannot be achieved in polarization 90°. In addition, in the Supplementary Movie 1, we show the effect of 360 degrees rotation of the RX polarizer, and we can see the glass depth varies accordingly, which once again proves the ability of the PSL 3D sensor.

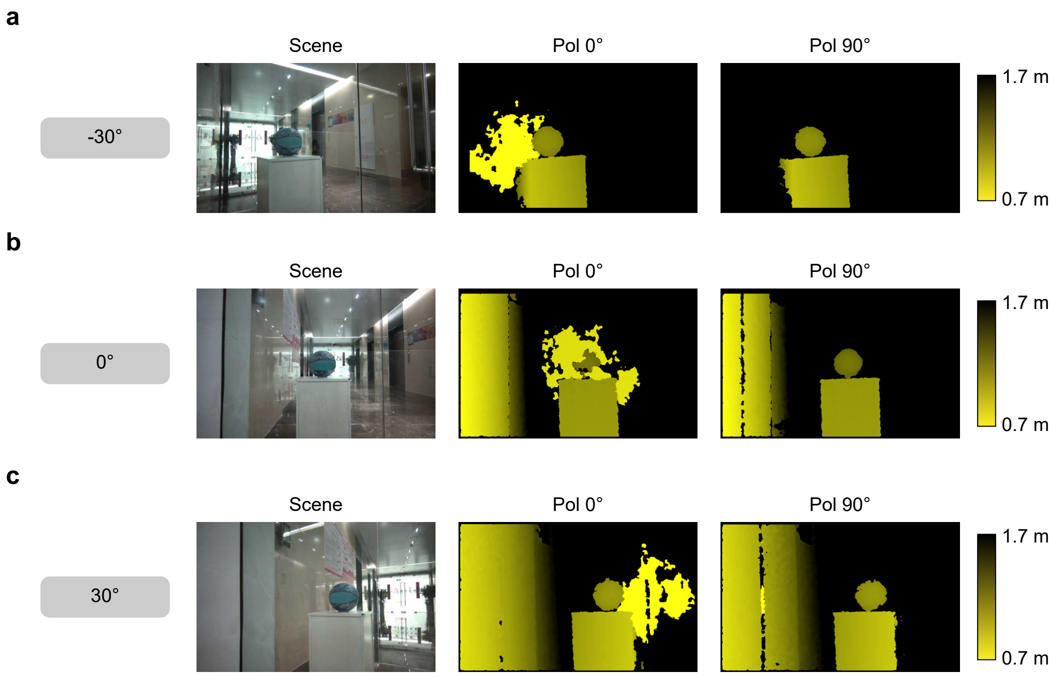

Figure S5: **Results of different incident angles of the PSL 3D sensor.** **(a)**-**(c)** are results for angles of −30°, 0°, 30°, respectively. The pictures of scenes are shown in the first column. The column of Pol 0° is the depth map when TX and RX are in the same polarization. The column of Pol 90° is the depth map when TX and RX are in the orthogonal polarization. The yellow-black colorbars apply to both second and third columns.

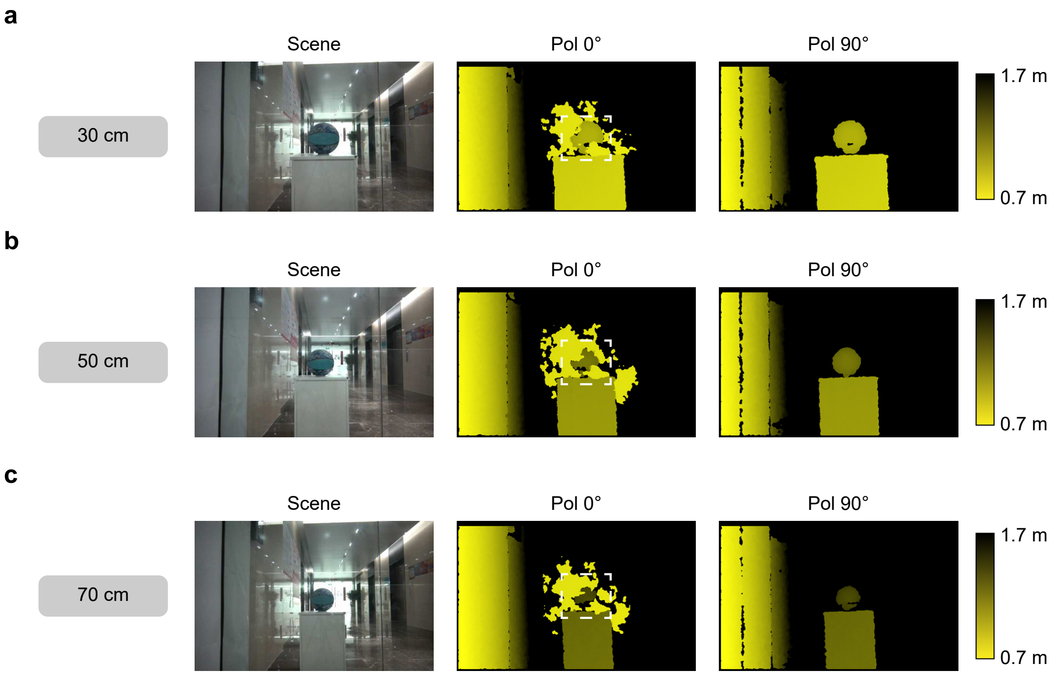

Figure S6: **Results of different distances of object behind glass. (a)-(c)** are results for distances of 30 cm, 50 cm, 70 cm, respectively. The pictures of scenes are shown in the first column. The column of Pol 0° is the depth map when TX and RX are in the same polarization. The column of Pol 90° is the depth map when TX and RX are in the orthogonal polarization. The yellow-black colorbars apply to both second and third columns.

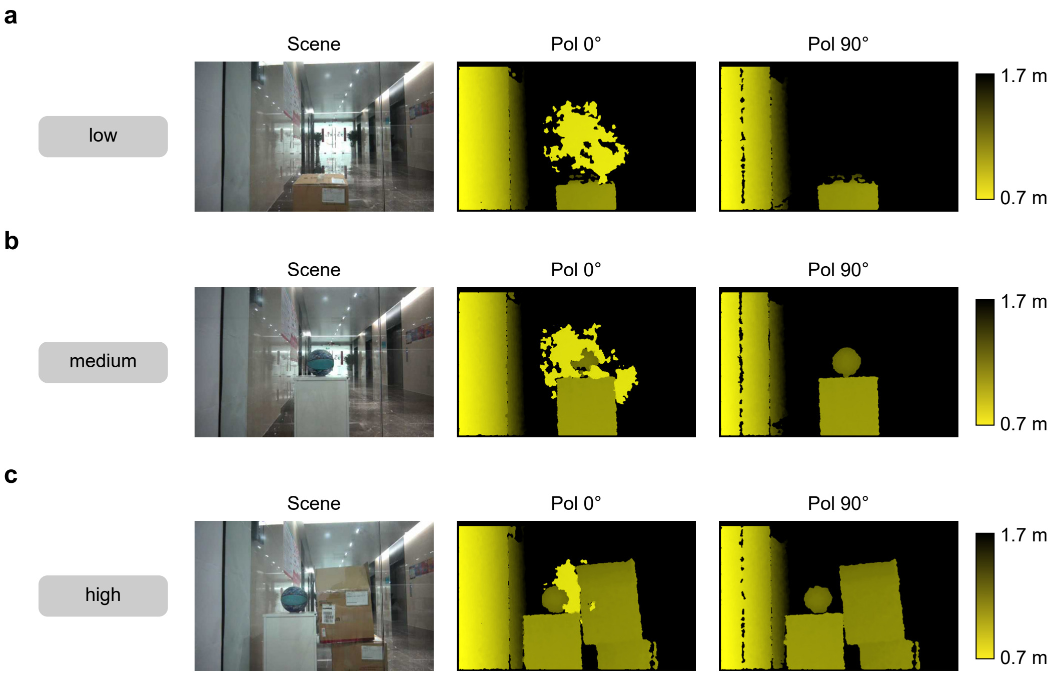

Figure S7: **Results of different densities of object behind glass. (a)-(c)** are results for density levels of small, medium

and large, respectively. The pictures of scenes are shown in the first column. The column of Pol 0° is the depth map when TX and RX are in the same polarization. The column of Pol 90° is the depth map when TX and RX are in the orthogonal polarization. The yellow-black colorbars apply to both second and third columns.

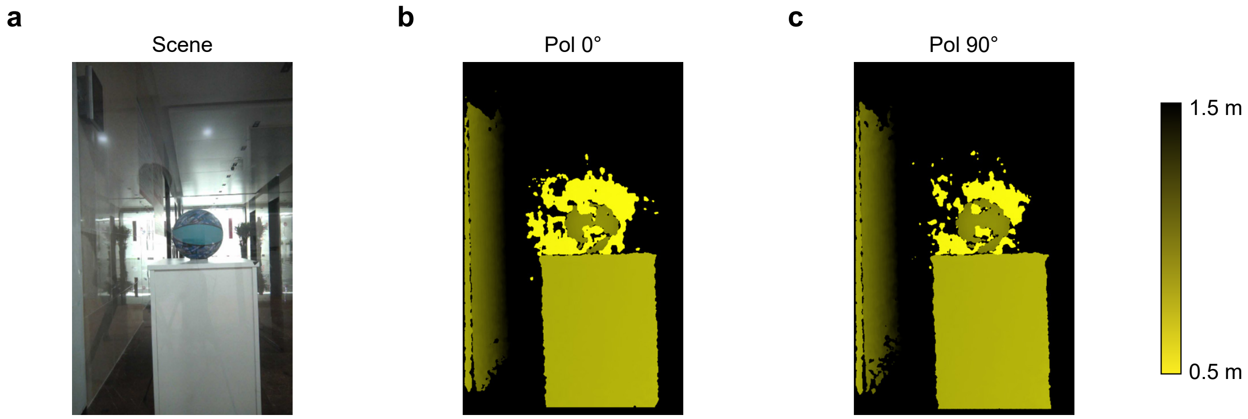

Figure S8: **Results of DBR-VCSEL-based sensor.** **(a)** The picture of the scene. **(b)** Depth map from Pol 0°. **(c)** Depth map from Pol 90°. The yellow-black colorbar apply to both **(b)** and **(c)**.

VI. Analysis on seeing against reflective noise.

In Fig. S9, we show another comparative experiment for detecting objects inside glass from the outside. In this scene, different from the normal incidence in Fig. 3b of the main text, we detect the glass scene in an angle (Fig. S9a). On the glass, there are reflections of external trees and hand. Inside the glass, there is a person to be detected. We can see that the reflective noise on the glass overlaps with the position of the person to be detected, which causes the stereo vision camera to fail to obtain the correct depth of the person. As we can see from Fig. S9b, the depth of the external tree and hand is incorrectly displayed on the depth map and blocks the depth information of the person inside, leaving only the leg in the depth map. But for the PSL 3D sensor, the correct depth information can be obtained through the polarization combination of TX and RX (Fig. S9c).

In the second case of seeing against reflective noise experiment in the main text, we compare the stereo vision camera and PSL 3D sensor in the scene of wall corner. In this case, although there are effects of reflection and multipath noise, PSL 3D sensor can obtain complete and correct corner depth and point cloud through the combination of TX and RX P polarization. In the following experiments, we continue to analyze the effects of TX and RX polarization combination in the same scenario.

In Fig. S10, we show the experimental results at the height of 24 cm, where Fig. S10a, S10c and S10e are the results of TX y polarization (pol y), and Fig. S10b, S10d and S10f are the results of TX x polarization (pol x). In Fig. S10a, we see that when RX has no polarization, the depth map will have holes in the corner due to the influence of reflective noise. Compared with the results of stereo vision camera in the main text, although there are holes, the angle between the floor and the wall remains at a right angle in the point cloud. When we use TX pol x (Fig. S10b), since both signal and noise are x-polarized, the holes in the red dot line are larger than in Fig. S10a. Since the reflective noise is in the x direction, when we use the combination of TX pol y and RX pol y, as shown in Fig. S10c, we can complete the missing part in Fig. S10a and obtain the complete and correct depth information. Similarly, even if the TX and RX directions are orthogonal (Fig. S10d), most of the noise can be filtered out when we use RX pol y, making the missing part in Fig. S10b complete. But when RX is in the x direction, it allows a lot of noise to go through, so the depth map at the corner is missing again (Fig. S10e). These holes are more severe when both TX and RX are in the x direction (Fig. S10f). Through the above comparisons, we can see that in the case of reflection and multipath noise, the combination of TX pol y and RX pol y can filter the noise

and enable a clear 3D information acquisition of the scene. Although TX is not always orthogonal to the noise, we can still determine the direction of noise and use RX to increase the SNR like demonstrated in Fig. S10d. The polarization characteristic of PSL 3D sensor make it superior in the case of detecting against the reflective noise.

Similar to Fig. S10, we show the results at the height of 40 cm in Fig. S11. Compared with those of Fig. S10, the impact of reflective noise is not so big at 40 cm, as shown in the dashed box in Fig. S11a. However, for TX pol x, there is still noise in the middle of the depth map (red dot line in Fig. S11b). Therefore, the combination of TX pol y and RX pol y in Fig. S11c still provides the best depth map information. Similarly, even if TX and RX are orthogonal, the RX pol y can still filter the noise in the middle (Fig. S11d). For Fig. S11e and S11f, because the polarization direction of RX and noise are both x, the depth information is missing and Fig. S11f is the worst situation because TX and RX are both pol x.

In Fig. S12, we show the results at the height of 56 cm. At this height, the reflective noise has little effect. As shown in Fig. S12a, with only TX pol y, the depth in the red dot line is already complete. For TX pol x, there will still be a small effect of reflective noise in the middle (blue dot line in Fig. S12b). As for the combination of TX pol y and RX pol y, it can get a complete depth map and point cloud as Fig. S12a, and will not be affected by the halving of the polarization power at the RX. In the setting of Fig. S12d, although the noise in the middle of the blue box is eliminated, there is some missing in the edge due to the orthogonal polarization (shown in the red box). Similarly, Fig. S12e will have this problem. As for Fig. S12f, it has the worst depth result because both TX and RX are set in the same direction as the noise. Therefore, in this scenario, the polarization combinations in Fig. S12a-S12c all work for depth detection. In future work, we can design a RX that receives both pol x and pol y at the same time, solving the problems in Fig. S12d and S12e.

In addition, we also test the ToF sensor in this scenario. As shown in Fig. S13, ToF sensor is unable to obtain the correct depth information at all heights. In detail, we can see that the floor part in the pointcloud (pointed by red dot box and solid arrow) penetrates into the plane because of the impact of noise while the PSL 3D sensor works well at all heights with its polarization settings.

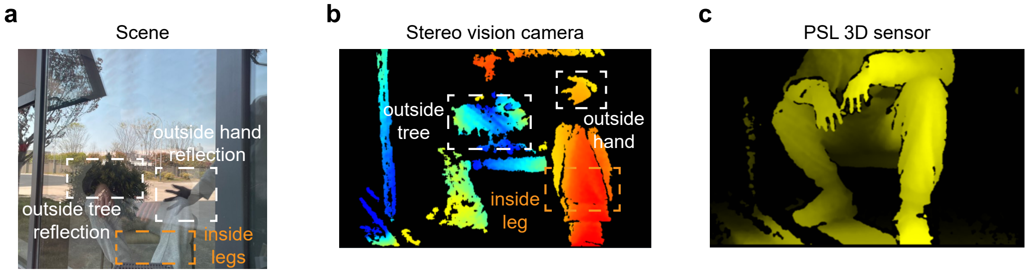

Figure S9: **Comparison on an outdoor glass scene.** **(a)**Scene of the experiment. **(b)** Depth map from the stereo vision camera. **(c)** Depth map from the PSL 3D sensor.

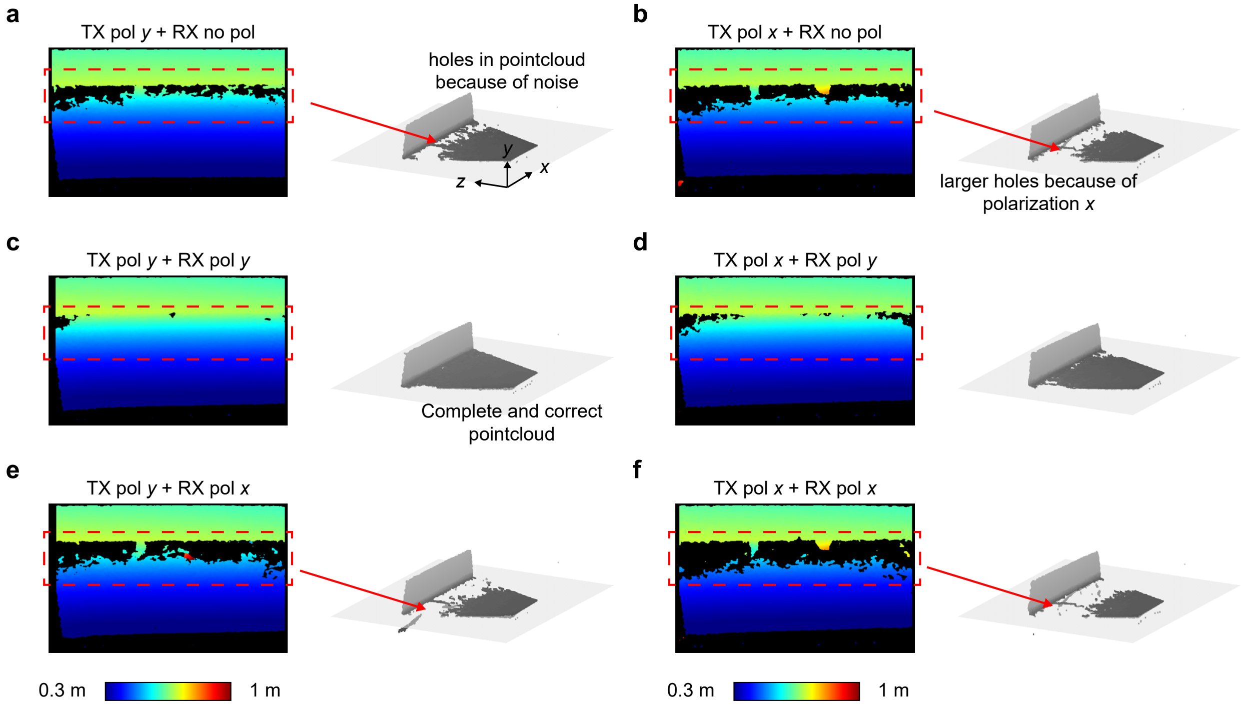

Figure S10: **Comparison at the height of 24 cm.** **(a)**-**(c)** are results of TX pol y and **(b)**-**(f)** are results of TX pol x. RX has no polarization in **(a)** and **(b)**, RX is pol y in **(c)** and **(d)**. RX is pol x in **(e)** and **(f)**.

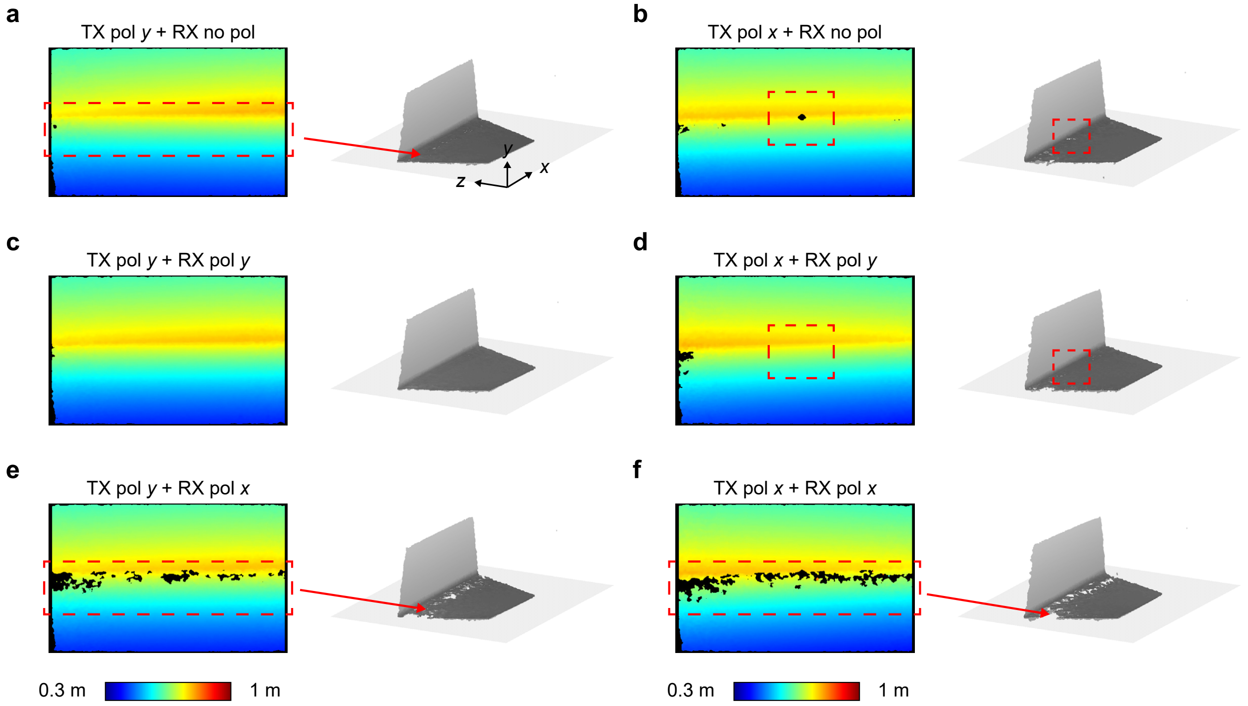

Figure S11: **Comparison at the height of 40 cm.** **(a)**-**(c)** are results of TX pol y and **(b)**-**(f)** are results of TX pol x. RX has no polarization in **(a)** and **(b)**, RX is pol y in **(c)** and **(d)**. RX is pol x in **(e)** and **(f)**.

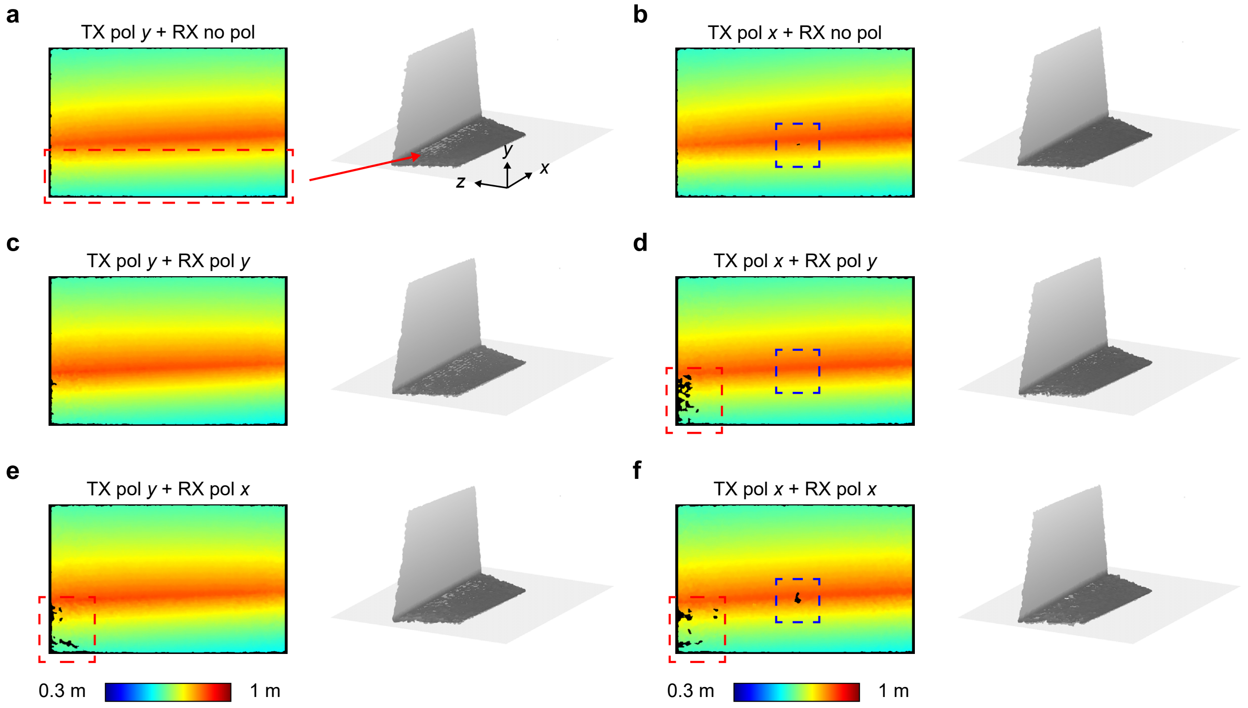

Figure S12: **Comparison at the height of 56 cm.** **(a)**-**(c)** are results of TX pol y and **(b)**-**(f)** are results of TX pol x.

RX has no polarization in **(a)** and **(b)**, RX is pol y in **(c)** and **(d)**. RX is pol x in **(e)** and **(f)**.

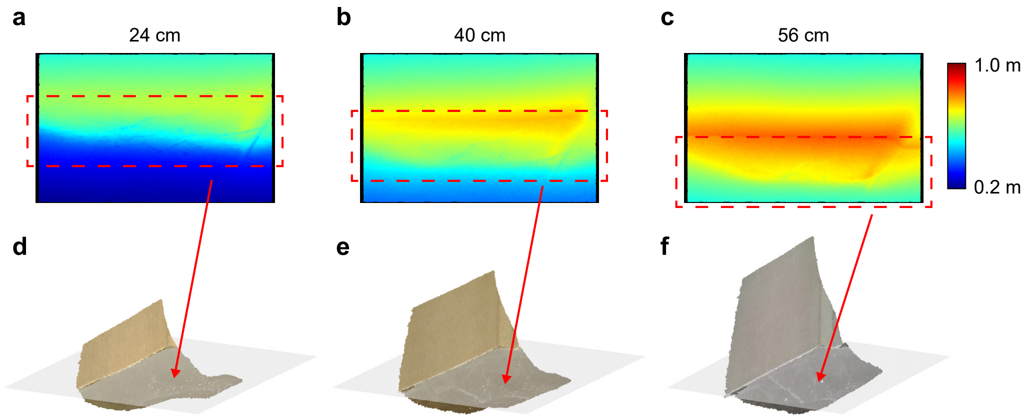

Figure S13: **Detecting results from ToF sensor.** **(a)**-**(c)** are depth maps at different heights. **(d)**-**(f)** are their corresponding point clouds. The jet colorbar applies to **(a)**-**(c)**.

VII. Detection and completion results

In this section, we display the other four scenes (Fig. S14). They include the balcony glass #2, the fish tank glass, the mirror and the front door glass. As for the balcony glass #2 scene, it is also a glass door which has reflections of the interior furniture and the glass inclines to the right. With polarization 0° and polarization 90° settings, we obtain two depth images of the scene. In polarization 0°, partial of the glass and the objects around it can be reconstructed. In polarization 90°, the glass part is eliminated, leaving those from diffuse reflection. Thus, in the same way, using the subtraction of two depth images and the glass boundary predicted from the RGB image of the scene, we are able to extract the glass region, which is highlighted in red in the fifth row. The extracted glass points are then used to fit and complete the reflective surface. The completed depth images are shown in the seventh row. Because the glass on the right is not on the same plane as that on the left, we only select the left glass for completion manually. The final comparison between the original point cloud and the completed point cloud is shown in the last two rows, where the new completed glass is colored in red. Similar to the balcony glass #1 scene in the main text, the new completed glass also matches well to the glass frame.

In the fish tank scene, the glass tank with water inside is placed at the left of the field of view, while on the right side there is a bucket and a cabinet. In polarization 0°, we can see that the upper part of glass is detected. In polarization 90°, the upper glass depth is eliminated due to the orthogonal polarization. Again, we combine with the glass boundary in the fourth row to extract the glass region, which is shown in the fifth row. Similarly, this extracted depth information is used for the following fitting and completion. As shown in the row of depth completion and the last row, the front face of the fish tank can be reconstructed precisely.

We also test our method in the mirror scene. In this situation, the PSL 3D sensor is placed in front of the mirror. On the mirror, there are reflections of the sensor and other indoor objects. Likewise, two depth images are obtained from two polarization settings. We can see that the depth of the mirror surface is also eliminated in polarization 90°. With the subtraction from two depth images and the mirror boundary, we acquire the mirror region in the fifth row. Then we complete the mirror inside the boundary and get the completion result. Because we cannot see through a mirror physically, the measured depth information which is caused by the reflection of the mirror is removed, as shown in the comparison in the last two rows. For a surface with high reflectance like mirror, the structured light will first reflect to the objects in front of this surface and next propagate

back to the RX in the same way, leading to the acquisition of the mirror images. But this will not affect the elimination of the mirror between two depth images. Therefore, our method is still able to detect and complete the mirror, making the correct reconstruction of the scene. Furthermore, it is different from the situation encountered by the stereo vision camera. For a stereo vision camera, on the one hand, it is unable to obtain and determine the depth of the reflective surface. On the other hand, whenever there is a reflection in either a mirror or glass, it will mistakenly consider the reflected image as a real object behind the surface.

In the last column, we show the front door glass with reflecting image of two persons, confirming the advantage of PSL 3D sensor in the glass scene once again. In this situation, two persons and the tripod are reflected images while the vase is a real object behind the glass. In polarization 0°, only the vase and partial glass are obtained. When it changes to polarization 90°, the glass part is eliminated, leaving the depth of the vase. Then subtraction is employed between two depth images to extract the glass region, combined with the glass boundary. Next, the glass region is fitted and used for completion. From the final result, we can see that the glass can be completed well and the vase behind the glass can also be reconstructed.

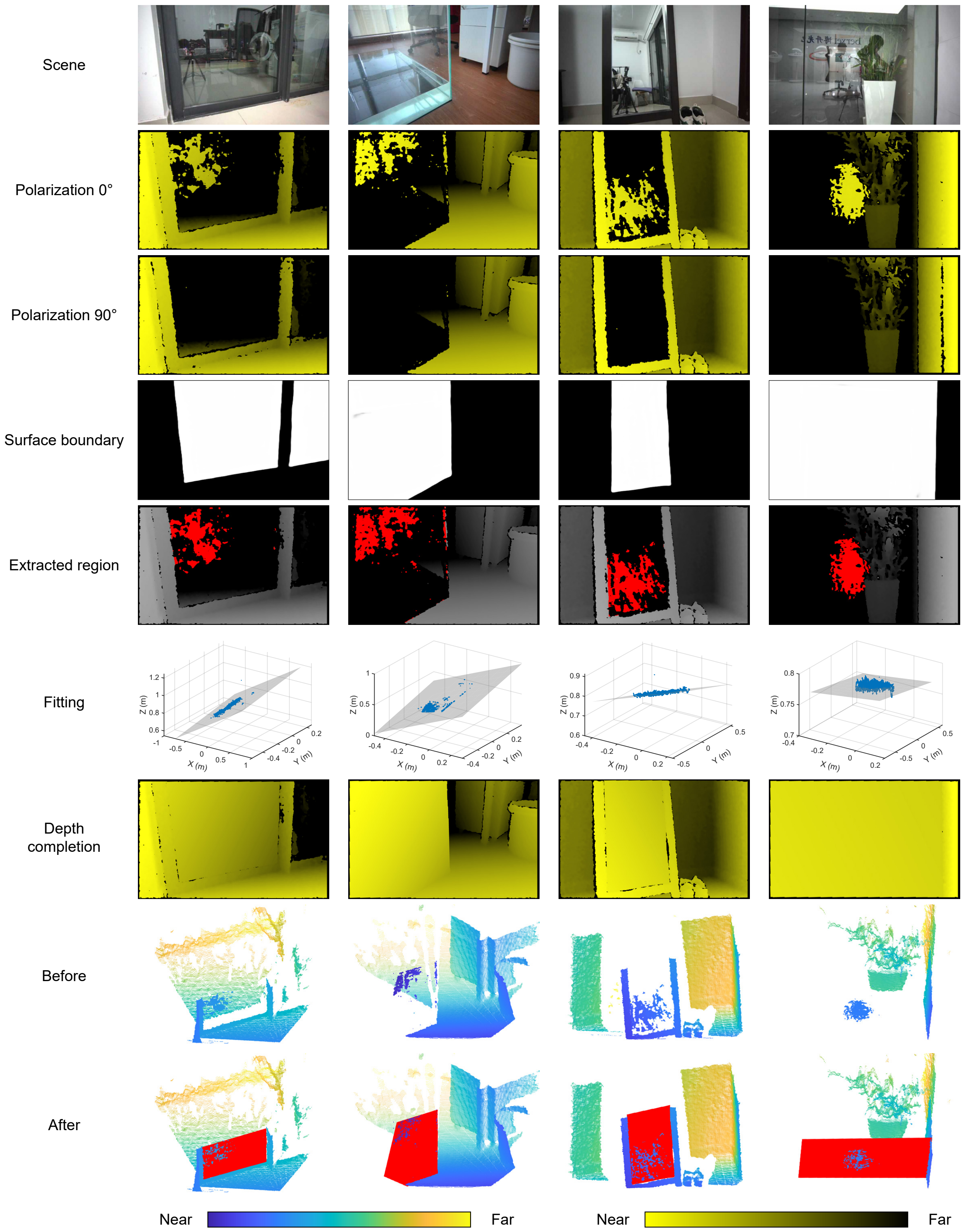

Figure S14: **Detection and completion results.** The results of the other four scenes are presented column-wise in this figure. The first row is the RGB image of each scene. The second and third row are the depth map from polarization 0°

and polarization 90° settings respectively. The fourth row is the predicted boundary using deep learning method. The fifth row is the extracted reflective surface region, which is obtained from the combination of depth image subtraction and predicted boundary. The sixth row shows the fitting of the extracted points in the world coordinate. The seventh row is the completed depth image. The original point cloud and the completed point cloud are shown in the last two rows. In the completed point cloud, the completed reflective surface is highlighted in red. The parula colorbar applies to the last two rows and the yellow-black colorbar applies to the rows of polarization 0°, polarization 90° and depth completion.

VIII. Detection area analysis

In the experiment, we find that the area of the reflective surface detected by the PSL 3D sensor will be affected by the cleanliness of the surface. In this section, we analyse the relationship between the level of cleanliness on the surface and the amount of depth information that can be extracted. As shown in Fig. S15a, we place the PSL 3D sensor at a distance of 0.8m directly facing the mirror. There are different kinds of natural dirt on the mirror surface, such as dust, water stain or fingerprints. We first set the PSL 3D sensor in pol 0°, that is, TX and RX are both in TM polarization, and then obtain the depth map of this mirror scene, the result of which is shown in the first row of Fig. S15b. We can see that due to the influence of surface particles, the specular reflection is redirected, and most of the reflected light can be received by the RX, thus obtaining a large area of mirror depth. This kind of reflection still maintains the original polarization direction, so when we set the RX in pol 90°, as shown in the second row of Fig. S15b, the mirror part is eliminated, while the reflection from the wall which has different polarized directions due to diffuse reflection is reserved in the depth map. Then we wipe the mirror surface with alcohol to gradually improve the cleanliness, and their depth results are shown in Fig. S15c, S15d and S15e. We can see that in pol 0°, the depth area of the mirror surface gradually decreases as the cleanliness increases and in pol 90°, the depth information belonging to the mirror surface will be eliminated. Fig. S15e is the result of the cleanest situation, which means there is only complete specular reflection and not off-specular reflection [3]. Hence in pol 0°, the mirror area that can be detected is very small, and its corresponding theoretical model is illustrated in Fig. S15f. As shown in Fig. S15f, the baseline distance between the TX and RX of PSL 3D sensor is b (b = 4cm in the PSL 3D sensor), and the distance from the PSL 3D sensor to the reflective surface is d (d = 80cm in this section). In the situation where the PSL 3D sensor faces the reflective surface, the imaging light path of complete specular reflection is shown by the blue solid line. According to the reflection law, the incident angle θ_{i1} is equal to the reflection angle θ_{r1}, so only an area with the size of s_1 can be received by the RX. In the situation of oblique illumination where the reflective surface has an inclined angle α, the imaging light path is shown by the grey solid line. Similarly, the incident angle θ_{i2} is equal to the reflection angle θ_{r2}, and only the area with the size of s_2 can be received by the RX. The size of s_1 and s_2 is basically equal to the area of TX. When d/b is large, s_1 or s_2 will appear relatively small, as shown in the first row of Fig. S15e.

However, in common scenes, these situations where there is only specular reflection are rarely

seen. Instead, a more suitable reflection model is shown in Fig. S15g. In this model, the projection range of TX is indicated by grey dot arrow lines and the receiving range of RX is indicated by orange dot arrow lines. As pointed by the blue solid arrow lines, the TX projects structured light onto the reflective surface and the reflected light will be affected by the reorientation of surface particles. In this situation, off-specular reflection dominates, which broadens the reflection lobe. Thus, in the receiving range, a larger area s can be detected by the RX. Furthermore, this reflection is not diffuse reflection and will still maintain the original polarized direction [3, 4], so when TX and RX have orthogonal polarized directions, the information belonging to the reflective surface will be completely eliminated. Benefitting from the off-specular reflection, for daily application scenarios, our PSL 3D sensor can extract enough depth information of the reflective surface and use it for the detection and completion applications, as demonstrated in Fig. 6 of the main text and Fig. S14 of the Supplementary Information.

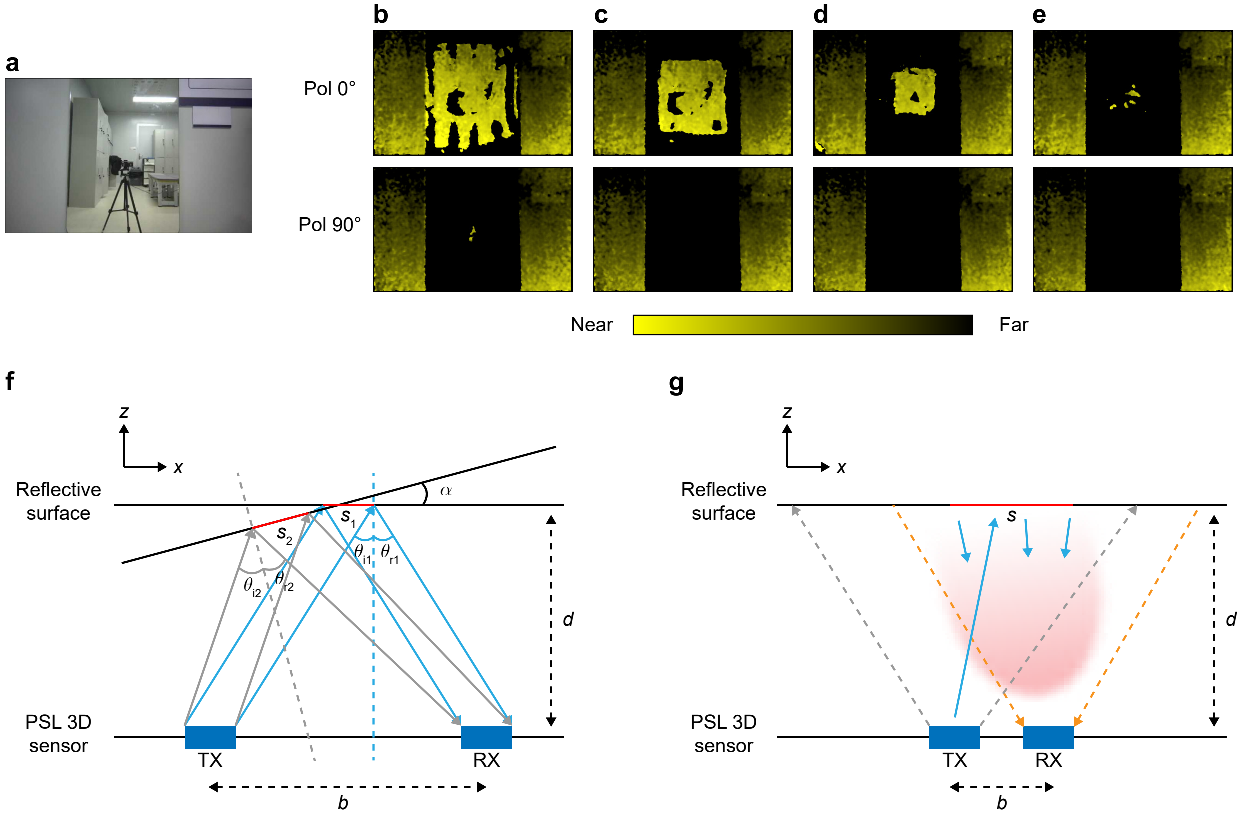

Figure S15: **Detection area analysis.** **(a)** The experimental scene for detection area analysis. **(b)-(e)** are the depth maps of the scenes with different degrees of cleanliness. The first row is the result of pol 0°. The second row is the result of pol 90°. **(f)** Light path model of complete specular reflection. **(g)** The reflection model for common scenarios.

IX. Working range of the PSL 3D sensor

In actual 3D applications, the glass scene is very complicated and cannot face our PSL 3D sensor every time. Therefore, we give the effective measurement range here. We fix the distance from the sensor to the flat glass door as 1 meter, and then measure the working range of the sensor by rotating it in the horizontal and vertical directions (Fig. S16a). Specifically, we take the number of depth points that belong to the glass when the sensor is at angle 0° as a benchmark, and then calculate the proportion of glass points relative to this benchmark at different angles. We take the angle at which the scale drops to 2% as the imaging limit.

In the horizontal direction, we measure 5 angles, $\{0°, 15°, 30°, 37.5°, 45°\}$. The depth maps of each angle are shown in the first row of Fig. S16b and their corresponding extracted glass regions are highlighted in the second row. Thus, we take the glass region of angle 0° as a benchmark and calculate the proportion of glass regions in other angles with respect to this benchmark. The proportion is shown in Fig. S16d, where the percentage drops to 2.66% at angle 45°. In the vertical direction, we also measure 5 angles, $\{0°, 7.5°, 15°, 22.5°, 30°\}$. With the same method, we show the results in Fig. S16c and Fig. S16e, where the percentage drops to 2.49% at angle 30°. Note that we only measure in the positive range, whereas it can expand to the negative range. Thus, the effective working range of our PSL 3D sensor is ±45° horizontally and ±30° vertically.

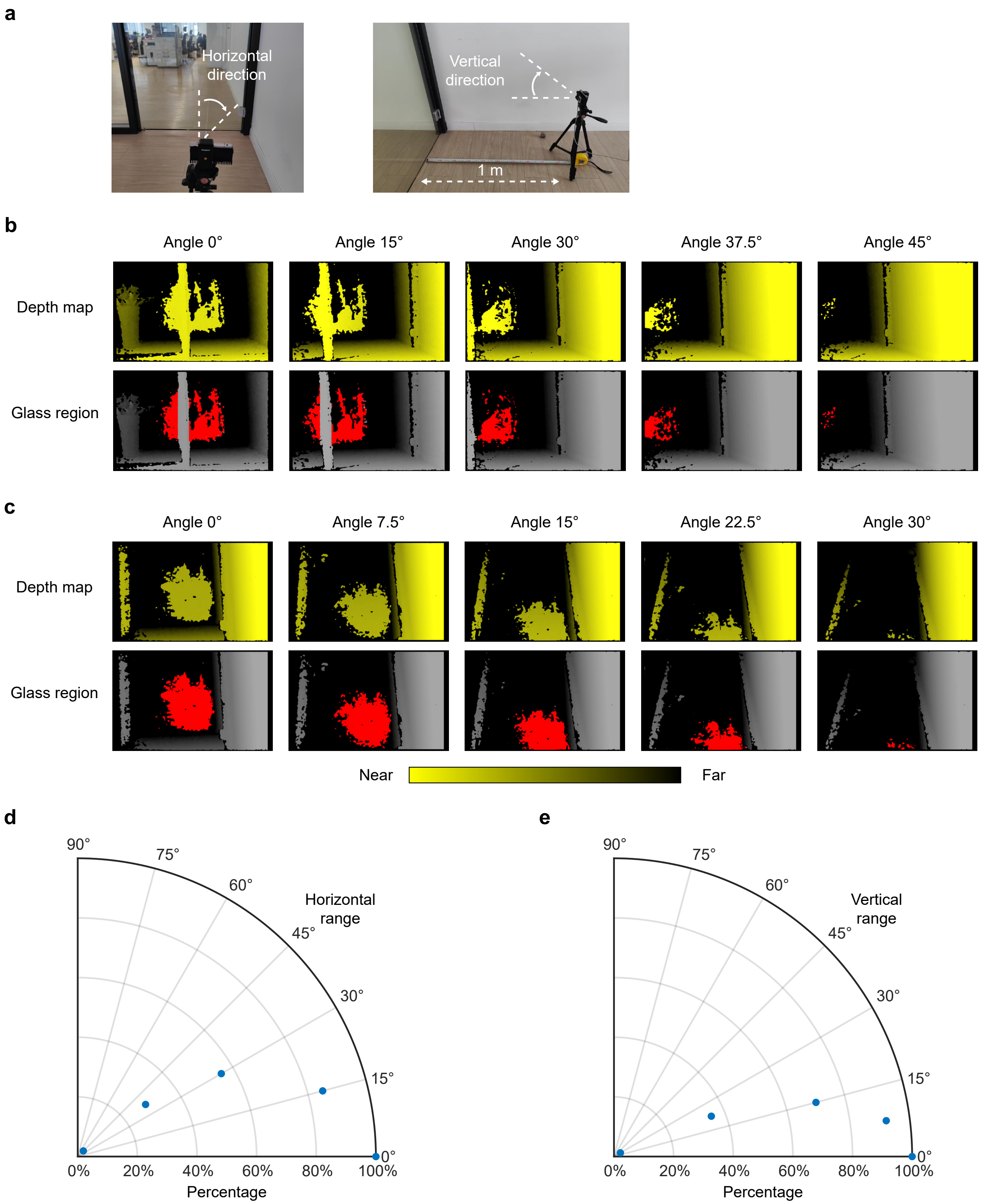

Figure S16: **Horizontal and vertical working range.** **(a)** Angle measurement setups of horizontal and vertical direction. **(b)** Measurement results of the horizontal range. **(c)** Measurement results of the vertical range. **(d)** The proportion of glass points in the horizontal direction. **(e)** The proportion of glass points in the vertical direction.

X. Panoramic reconstruction of a fish tank

All the detection and completion experiments in the main text are captured in the single view. If we want to reconstruct a complete 3D shape of an object, we need to employ our method in various angle. In this section, we have achieved the 360° panoramic reconstruction of a fish tank scene using multi-angle fusion. Specifically, we capture four sides of the fish tank using the PSL 3D sensor, as shown in the first row of Fig. S17a. By setting the polarization of the PSL 3D sensor to 0° and 90°, we obtain depth images at two different angles, as illustrated in the second and third rows of Fig. S17a. Consistent with previous experiments, when polarization is 0°, the PSL 3D sensor can reconstruct the glass part of the fish tank and the surrounding objects. When polarization is 90°, it can eliminate the glass part and retain only the surrounding diffuse components. Combined with the glass boundary in the fourth row, we then extract, fit and complete the glass, as shown in the fifth, sixth and seventh rows of Fig. S17a, respectively. Finally, by combining the reconstructions of the four sides of the fish tank with the ground as the reference plane, we use CloudCompare [5] to fuse the multi-angle data and achieve the 360° panoramic reconstruction of the fish tank scene, as shown in the 3D color and grayscale schematics in Fig. S17b and S17c, respectively.

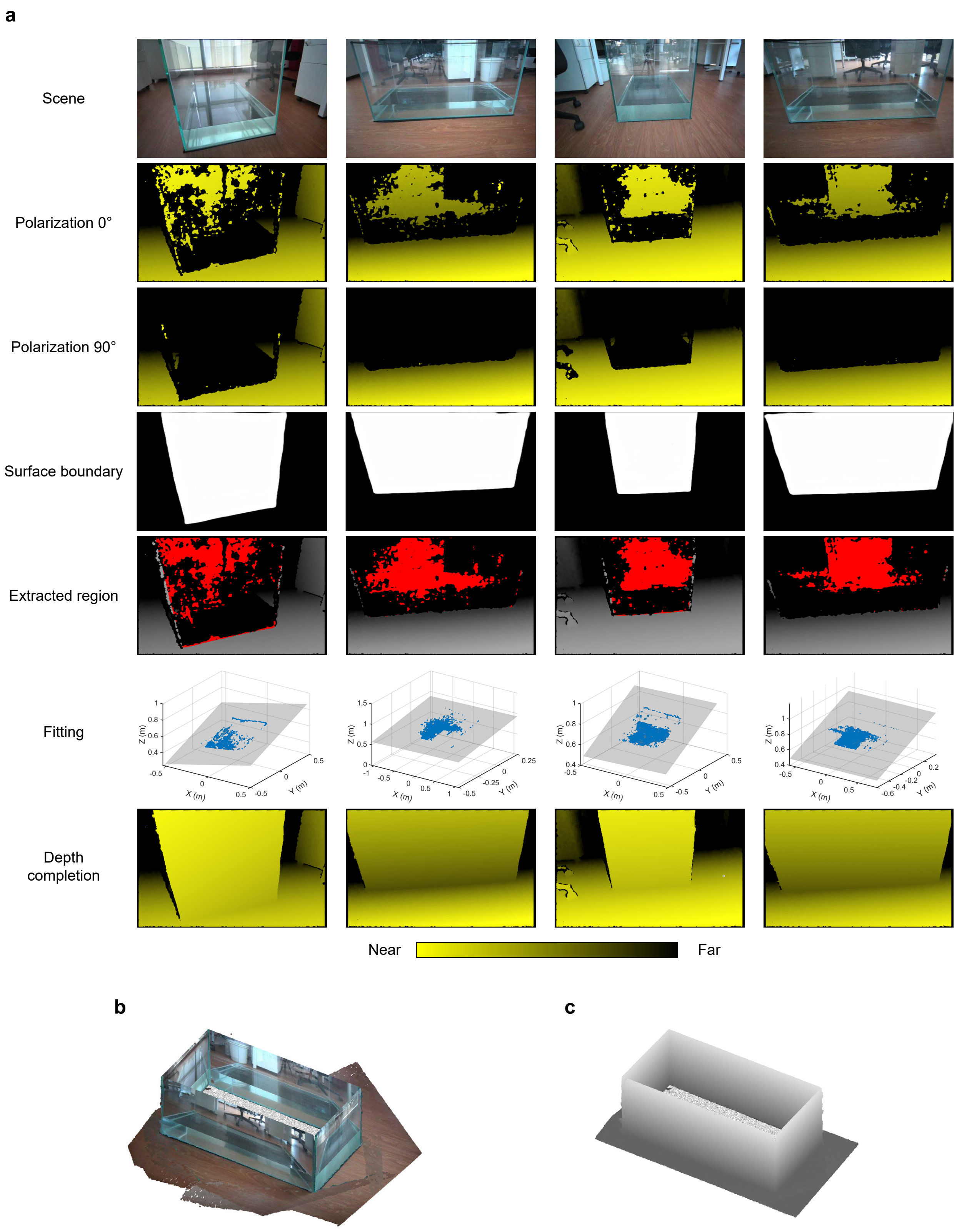

Figure S17: **Panoramic reconstruction of a fish tank.** **(a)** Detection and completion results of four sides of the fish tank. **(b)** and **(c)** are the panoramic reconstruction of the fish tank, rendered with color and in grayscale, respectively.

XI. Detection and completion result of scene with glass and mirror

In this section, we experimentally verify the reconstruction ability of the PSL 3D sensor when there is another strongly reflective object (such as a mirror) behind the glass. In this scene, the PSL 3D sensor is placed 1 m in front of the glass door, and a rectangular mirror and a cabinet are placed 0.5 m behind the glass door, as shown in Fig. S18a and S18b. We use the PSL 3D sensor to obtain two depth images at polarization 0° and 90°, as shown in Fig. S18c and S18d. At polarization 0°, we are able to obtain the depth of the front glass (red dot line) and the back mirror (white dot line). At polarization 90°, these two parts are eliminated simultaneously and only the surrounding diffuse components are remaining. After applying subtraction, the depths of the glass and mirror are both retained, as shown in S18f. But our main purpose is to identify the front obstacle. Hence, we can filter out the reflection from the back, making it successful to determine the front glass area, which is demonstrated in Fig. S18g. The final completed result is shown in Fig. S18i. As seen from the error map in Fig. S18j, our method can achieve reconstruction with high precision of 2 mm on the average.

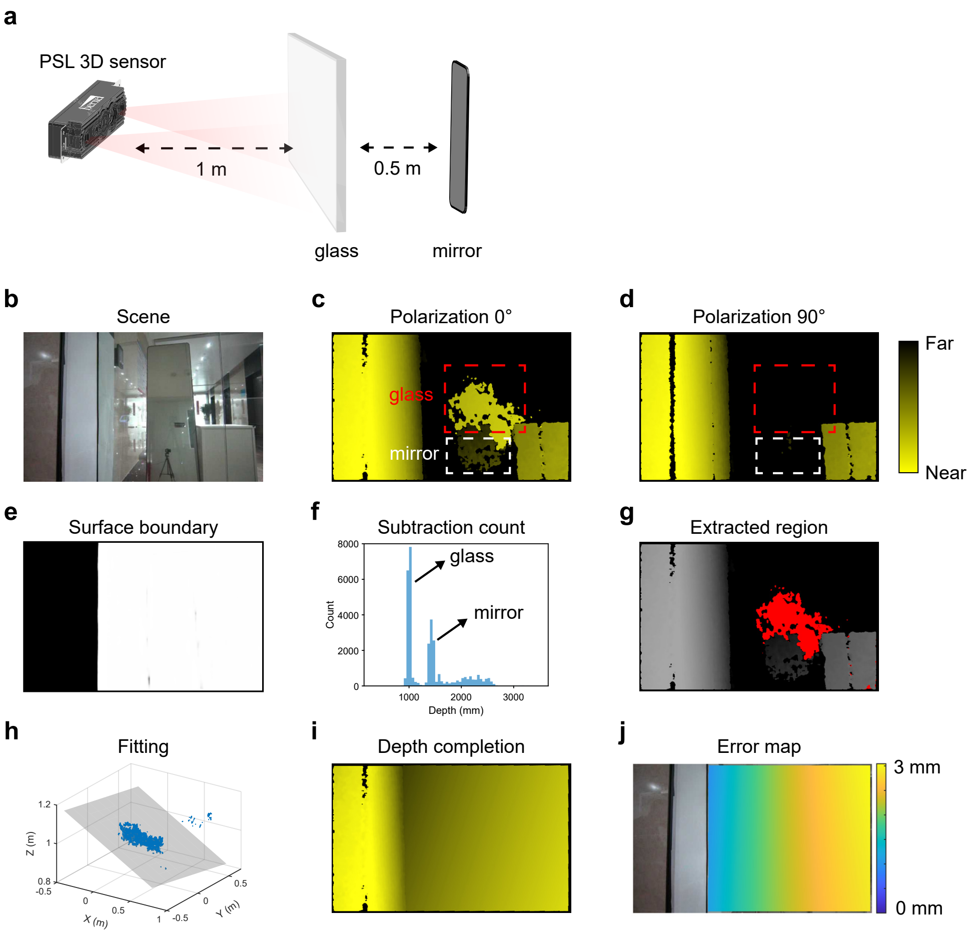

Figure S18: **Detection and completion result in scene with glass and mirror.** **(a)** Scheme of the experiment. **(b)** RGB image of the scene. **(c)** Depth map at polarization 0°. **(d)** Depth map at polarization 90°. **(e)** The predicted boundary. **(f)** The subtraction count. **(g)** The extracted reflective surface region. **(h)** Fitting of the extracted points in the world coordinate. **(i)** The completed depth image. **(j)** The calculated error map.

XII. Comparision with ToF sensor on detection and completion of reflective surfaces

In this section, we show the shortcoming of ToF sensor in the task of detecting and completing glass. In this comparison, we place the sensor 51 cm directly in front of the glass (Fig. S19a). Since the purchased ToF sensor (Orbbec Femto W) has been integrated together, we cannot replace the source with the HCG VCSEL, so we add polarizers to its TX and RX for testing. As shown in Fig. S19b, TX is set to pol 0° and RX is set to two directions. We also test the PSL 3D sensor in the same scene. The RGB pictures of the scene obtained by the ToF sensor and PSL 3D sensor are displayed in Fig. S19c and Fig. S19d respectively, where the diffuse sticker on the glass is denoted by orange dot line.

In this experiment, the results of ToF sensor are shown in Fig. S19e to S19h. As seen from Fig. S19e, the area of glass that can be detected is very small (denoted by white dot line), even though neither TX nor RX has a polarizer added. In addition, the depth of the sticker on the glass is denoted by orange dot line. When we add the polarizer of pol 0° to TX, the glass and sticker can be measured (Fig. S19f). When we add a polarizer of 0° to the RX, both the glass and sticker can still be detected because of the same polarization of TX and RX (Fig. S19g). Although the tree and wall behind are absent because of the occlusion, these are irrelevant factors, and we only need to focus on the parts of glass and sticker. When we place the polarizer of RX in pol 90°, the glass part is eliminated in the depth map because its reflection maintains the same polarization, while the diffuse sticker is detected in the depth map (Fig. S19h).

As for the PSL 3D sensor, as shown in Fig. S19i, we get a wide range of glass depth when we set it to polarization 0°. If we change to polarization 90° (Fig. S19j), the glass part can be removed, while the sticker on the glass is retained because of the diffuse reflection.

Although the ToF sensor can also add polarization characteristics to detect and eliminate the glass depth, it cannot obtain enough glass depth information because the depth calculation method is different from that of structured-light-based method. Therefore, PSL 3D sensor remains a powerful tool for the task of detecting and completing reflective surfaces.

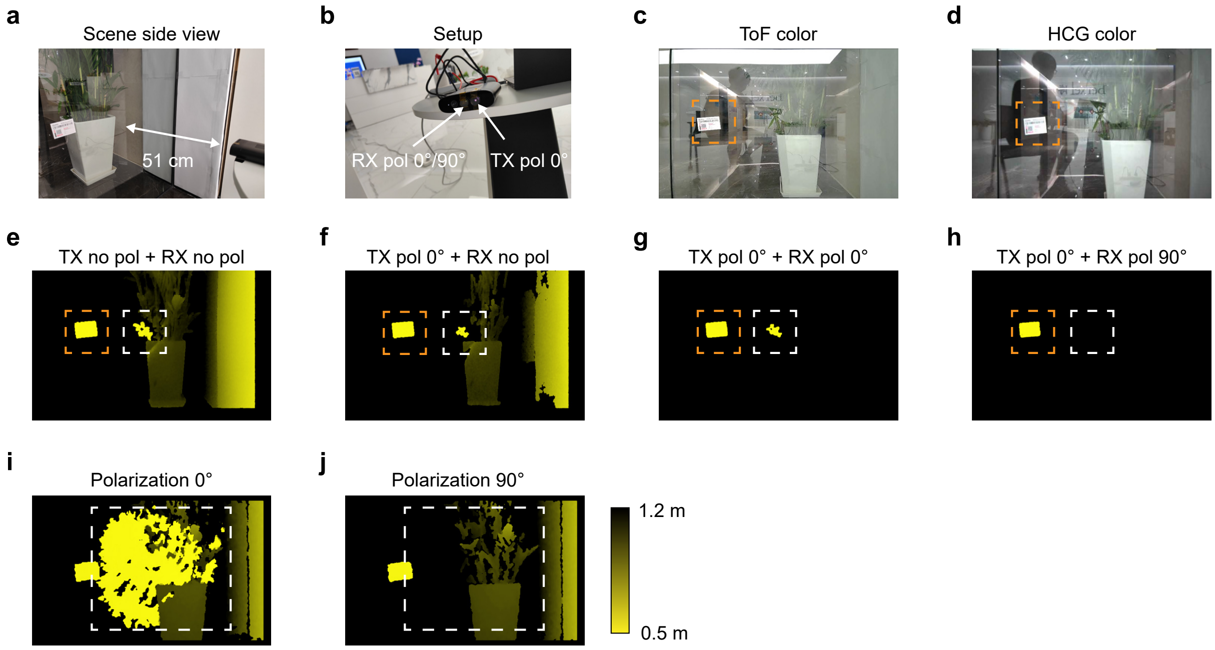

Figure S19: **Comparision with ToF sensor.** **(a)** Side view of the scene. **(b)** Setup of the ToF sensor. **(c)** Color image of the scene from the ToF sensor. **(d)** Color image of the scene from the PSL 3D sensor. **(e)**-**(h)** are depth maps from the ToF sensor, where their experimental settings are shown on the top of each figure. **(i)**-**(j)** are the results from the PSL 3D sensor.

XIII. Calculating the depth of the reflective surface

In this section, we use the scene in Fig. S5b as an example to show how the glass depth is calculated. We extract the infrared images received at the RX and present them at Fig. S20b and S20c. As we can see from Fig. S20b, at polarization 0°, we can obtain dot pattern reflected from the glass (denoted by the white dot line). Then we can calculate the depth of the glass according to the offset relationship between these received speckles and the original reference pattern, and the depth result is shown in Fig. S20d. Because these reflected speckles remain the original polarization, when we set to polarization 90°, the glass speckles are removed in the infrared image (Fig. S20c), and also the information that belongs to the glass in the depth image (Fig. S20e).

In addition, we illustrate the common triangulation method in Fig. S20f. In this scheme, the distance to the reference plane L, the baseline b between TX and RX, the imaging focal length f are known parameters. The point C is the detecting point, whose depth is our target. Due to the displacement effect of point C, the original reference point F on the imaging plane is now moved to G. If we calculate the displacement distance of FG, we can get the depth based on similar triangle relation,

$$\text{Depth} = \frac{L \times b \times f}{f \times b + \overline{FG} \times L} \tag{2}$$

Thus, the depth of the glass can be calculated with this method based on the received speckles.

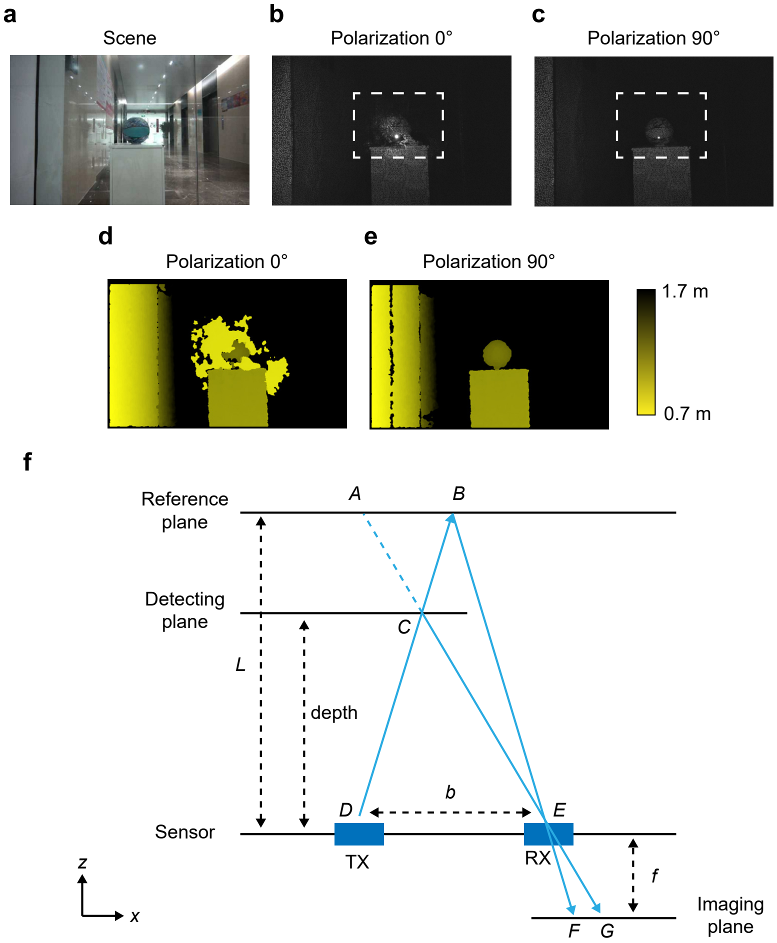

Figure S20: **The method of calculating depth.** **(a)** Picture of the scene. **(b)** Infrared image at polarization 0°. **(c)** Infrared image at polarization 90°. **(d)** and **(e)** are the corresponding depth maps. **(f)** Principle of the triangulation method.

Supplementary References

[1] Huang, M. C., Ye, Z. & Chang-Hasnain, C. J. A surface-emitting laser incorporating a high-index-contrast subwavelength grating. *Nature Photonics* **1**, 119–122 (2007).

[2] Huang, M. C., Ye, Z. & Chang-Hasnain, C. J. Polarization mode control in high contrast subwavelength grating vcsel. In: *2008 Conference on Lasers and Electro-Optics and 2008 Conference on Quantum Electronics and Laser Science*, 1–2 (2008).

[3] Torrance, K. E. & Sparrow, E. M. Theory for off-specular reflection from roughened surfaces. *J. Opt. Soc. Am.* **57**, 1105-1114 (1967).

[4] Wolff, L. B., Nayar, S. K. & Oren, M. Improved diffuse reflection models for computer vision. *International Journal of Computer Vision* **30**, 55–71 (1998).

[5] CloudCompare (version 2.10.alpha) [GPL software]. (2023). Retrieved from http://www.cloudcompare.org/
