# Supplementary material for: Polarization structured light 3D depth image sensor for scenes with reflective surfaces
Source: Nat Commun. 2023 Oct 27;14:6855. doi: 10.1038/s41467-023-42678-5 (PMC10611810; doi:10.1038/s41467-023-42678-5)
Supplement: Supplementary file 3 — Description of Additional Supplementary Files [file 41467_2023_42678_MOESM3_ESM.pdf]

Description of Additional Supplementary Files:

Supplementary Movie 1: This movie shows the depth map of the front door glass scene (the fourth column of Supplementary Fig. S14) as the polarizer of the RX is rotated from 0 degree clockwise for a full range of 360. It clearly demonstrates how the reflection from the glass panel can be eliminated by varying the polarizer angle. The reflection changes from being strong (0 degree) to being entirely eliminated (90 degree), and back to strong (180 degree) etc.
